# Early Evaluation of an Ultra-Portable X-ray System for Tuberculosis Active Case Finding

**DOI:** 10.3390/tropicalmed6030163

**Published:** 2021-09-04

**Authors:** Luan Nguyen Quang Vo, Andrew Codlin, Thuc Doan Ngo, Thang Phuoc Dao, Thuy Thi Thu Dong, Huong Thi Lan Mo, Rachel Forse, Thao Thanh Nguyen, Cong Van Cung, Hoa Binh Nguyen, Nhung Viet Nguyen, Van Van Nguyen, Ngan Thi Tran, Giang Hoai Nguyen, Zhi Zhen Qin, Jacob Creswell

**Affiliations:** 1 Friends for International TB Relief, Ha Noi 100000, Vietnam; andrew.codlin@tbhelp.org (A.C.); thuy.dong@tbhelp.org (T.T.T.D.); rachel.forse@tbhelp.org (R.F.); 2 IRD VN, Ho Chi Minh City 700000, Vietnam; ngodoanthuc@gmail.com (T.D.N.); thangdao9894@gmail.com (T.P.D.); huong.mo@ird.vn (H.T.L.M.); trannganhsph@gmail.com (N.T.T.); giang.nguyen@ird.vn (G.H.N.); 3 Pham Ngoc Thach Hospital Quang Nam, Tam Kỳ 560000, Vietnam; thaomaidao@yahoo.com.vn; 4 National Lung Hospital, Ha Noi 100000, Vietnam; vancong13071964@gmail.com (C.V.C.); nguyenbinhhoatb@yahoo.com (H.B.N.); vietnhung@yahoo.com (N.V.N.); 5 Department of Health Quang Nam, Tam Kỳ 560000, Vietnam; nhivan6@gmail.com; 6 Stop TB Partnership, 1218 Geneva, Switzerland; zhizhenq@stoptb.org (Z.Z.Q.); jacobc@stoptb.org (J.C.)

**Keywords:** tuberculosis, active case finding, X-ray, ultra-portable, handheld, vulnerable populations

## Abstract

X-ray screening is an important tool in tuberculosis (TB) prevention and care, but access has historically been restricted by its immobile nature. As recent advancements have improved the portability of modern X-ray systems, this study represents an early evaluation of the safety, image quality and yield of using an ultra-portable X-ray system for active case finding (ACF). We reported operational and radiological performance characteristics and compared image quality between the ultra-portable and two reference systems. Image quality was rated by three human readers and by an artificial intelligence (AI) software. We deployed the ultra-portable X-ray alongside the reference system for community-based ACF and described TB care cascades for each system. The ultra-portable system operated within advertised specifications and radiologic tolerances, except on X-ray capture capacity, which was 58% lower than the reported maximum of 100 exposures per charge. The mean image quality rating from radiologists for the ultra-portable system was significantly lower than the reference (3.71 vs. 3.99, *p* < 0.001). However, we detected no significant differences in TB abnormality scores using the AI software (*p* = 0.571), nor in any of the steps along the TB care cascade during our ACF campaign. Despite some shortcomings, ultra-portable X-ray systems have significant potential to improve case detection and equitable access to high-quality TB care.

## 1. Introduction

Tuberculosis (TB) is a curable disease, yet 1.5 million people die of TB each year [1]. TB is among the deadliest diseases caused by a single infectious agent, second only to SARS-CoV-2. While the COVID-19 pandemic has captured the world’s attention, its deleterious consequences have set back global efforts to end TB by 12 years [2], with an estimated incremental 400,000 deaths from TB occurring due to lack of access to quality care [3]. A vital strategy to ending TB prior to the COVID-19 pandemic was active case finding (ACF) through systematic screening for TB [4]. This strategy has further risen in importance due to pandemic-related social distancing and the resulting decreases in health-seeking behaviors and TB diagnoses [5,6,7]. Concordantly, the need to optimize the use of available tools to bend the curve towards ending TB will be paramount between now and the arrival of a TB vaccine [8,9,10]. A proven tool for TB screening with continued room for optimization has long been chest radiography, given its high sensitivity and utility as a rule-in test [11].

X-rays were discovered by Wilhelm Conrad Roentgen in 1895 [12] with the first generation X-ray system of Hoffmans and van Kleef following circa 1896 [13]. Chest X-ray (CXR) was considered a revolutionary advancement, and pulmonary TB became one of the most frequent radiologic diagnoses of that period despite use being limited overall [14]. After the first World War, a steep drop in TB mortality led to hope of disease eradication. Between 1930 and 1960, mass radiography became the strategy of choice for TB ACF. Particularly in the pre-chemotherapy period, mass radiography helped to reduce mortality by detecting individuals in earlier stages of disease progression, isolating sources of infection and identifying vulnerable persons eligible for intensified follow-up [15]. With improvements in mass miniature radiography, large-scale, population-wide screening with CXR took place among a broad range of target groups, buoyed by the success in identifying people early and in greater numbers than could be expected with earlier techniques [15]. Throughout this period, mass screening was taken up across the US and much of Europe.

However, CXR was not part of efforts to combat the disease in many high TB burden countries as the technology was, and remains until this day, prohibitively expensive with the cost borne largely by patients [16]. In high TB burden countries, the use of microscopy took hold beginning in the 1960s based on several pivotal studies from India and other Asian settings showing the utility of smear microscopy to identify people with TB who had a prolonged cough [17,18,19]. As verbal screening was cheap and easy, and microscopy could detect large numbers of people at a low cost, the WHO recommended against the use of large-scale CXR screening in 1974 while promoting microcopy. This trend was formalized in the WHO’s DOTS strategy in the early 1990s, which for decades actively dissuaded the use of CXR in TB, except for clinical diagnosis after multiple negative microscopy tests [20,21]. However, gaps in the DOTS strategy, such as insufficient consideration of children, people with smear-negative disease and people with HIV, as well as a paradigm shift from TB control towards TB elimination, have similarly fueled a departure from the passive approaches and return towards active screening for TB [22]. In step with the renewed attention in active screening was the rekindled interest in optimizing the utilization of CXR [23].

Specifically, CXRs have been increasingly used as a triage or screening tool instead of a diagnostic tool, as findings from modern prevalence surveys demonstrated that up to 60% of people with TB do not report the classical symptoms [24,25]. This transition was greatly aided by the digitization of X-ray and development of multi-layer detectors [26]. Digital radiography (DR) is characterized by high-quality images, simplified storage and archiving, lower marginal cost and reduced radiation exposure [27]. Newer software developments leverage artificial intelligence (AI) to enable unbiased visual analysis and interpretation without the need for highly trained human readers, who can be scarce in many settings, representing another enticing value proposition of CXR with AI [28]. Meanwhile, technological advances in the hardware have shrunk the physical properties and radiologic emissions to a fraction of past X-ray systems. The newest systems, labeled as ultra-portable or handheld X-ray systems, have compacted X-ray technology to the point where remote locations and home-based screening are now considered safe and feasible with units that can be hand carried.

Currently, there are at least three ultra-portable X-ray systems commercially available: the MINE 2 (HDT; Gwangju, Republic of Korea), Xair (FDR XD2000; Fujifilm Corporation; Tokyo, Japan) and Delft Ultra (Delft Imaging Systems, The Netherlands) systems. The Delft Ultra and Xair systems further include integration with software platforms for AI-supported interpretation of CXR. A major concern is that handheld X-ray devices emit lower doses of radiation, which in theory compromises image quality, and subsequently could impair TB case detection yields. The advantage is lower exposure to leakage doses and improved radiation safety for participants and healthcare workers. To help clinicians and TB program managers assess the potential benefits and disadvantages of these novel ultra-portable X-ray systems, we present results from a systematic performance evaluation of an ultra-portable X-ray system and share early implementation experiences from using the ultra-portable X-ray system in community-based TB ACF campaigns among remote populations.

## 2. Materials and Methods

### 2.1. Study Design

This was a cross-sectional study to assess the performance an ultra-portable X-ray system for TB ACF, measured by radiation exposure, image quality and case detection yields in comparison with facility- and community-based radiographic reference systems.

### 2.2. Study Objectives

The first objective was to conduct an ‘in vitro’ evaluation of the emission levels and standard operating parameters for posteroanterior (PA) chest radiography of an ultra-portable, battery-powered X-ray system (Fujifilm Xair), and compare its image quality with a conventional, stationary radiographic reference system (Quantum E7252; Carestream Health; NY, USA). Both systems used their manufacturer-specific detector sensors.

The second objective was to perform an ‘in vivo’ deployment of the ultra-portable X-ray system in community-based TB ACF campaigns and compare image quality, abnormality rate and TB case detection yields of the ultra-portable, battery-powered X-ray system compared with a community-based radiographic reference system, consisting of a semi-portable X-ray generator that required a continuous power connection (PXP-60HF; Poskom; Republic of Korea). For the field deployment, the ultra-portable and reference generators used the Fujifilm Xair flat-panel DR detector (D-EVO II G35) for capturing and processing the X-ray images.

### 2.3. Study Setting

The ‘in vitro’ evaluation was conducted in the radiology department of the National Lung Hospital (NLH) in Hanoi, Viet Nam. This department employs 14 radiologists and 28 X-ray technicians and is equipped with 9 stationary and mobile X-ray devices. The radiology department provides radiography services to an average of 600 persons per day, including 350 chest X-rays. Of these, the proportion of persons with TB-related abnormalities is approximately 10%.

The ‘in vivo’ deployment occurred in two remote areas of Viet Nam: a mountainous district (Phuoc Son) and an island (Tan Hiep) of Quang Nam province. Phuoc Son, located near the Viet Nam/Laos border, had a population of 26,337 in 2020 and notified 56 people with all forms of TB. Tan Hiep, located 8 km from the coast of Hoi An city, had a population of 2,091 in 2020, with a population density of 130/km^2^, and notified 3 people with all forms TB.

### 2.4. Ultra-Portable X-ray System

The ultra-portable X-ray system employed in this study consisted of two main components: (1) the X-ray generator and (2) the flat-panel detector. High-level specifications for both components as described in the user manual are provided below.

The X-ray generator was the Fujifilm Digital Radiography (FDR) Xair XD2000 system (Xair; Fujifilm Corporation, Japan). The generator is battery powered and has an output power of 450 W at 90 kilovolts (kV) and 5 milliampere (mA) and an exposure time range of 0.04–0.5 s. The weight of the device is 3.5 kg (kg) with dimensions of 30.1 cm (W) × 25.7 cm (D) × 14.4 cm (H).

The flat-panel detector was the multi-layer FDR DR-ID1201SE (D-EVO II G35; Fujifilm Corporation, Japan). The exposure size is 42.7 cm × 35.1 cm with a reading grayscale level of 16 bit/pixel and a pixel pitch of 150 μm. The detector is equipped with integrated image processing software that reduces the pixel pitch from 150 to 100 μm. X-ray images can be transmitted through a wired connection or wirelessly, and the detector has built-in storage for up to 100 images.

### 2.5. Participant Eligibility and Recruitment

Persons above 15 years of age attending the NLH radiology department and indicated for a CXR, or those attending the community-based CXR screening campaigns, were eligible for participation. We excluded persons contraindicated for CXR such as pregnant women and those declining to participate in the study.

Participants of the ‘in vitro’ study were consecutively recruited at the NLH from 15–26 March 2021. Community screening events were held from 29 March to 6 April 2021 in Phuoc Son and 9–14 April 2021 in Tan Hiep for the ‘in vivo’ study. The ultra-portable X-ray system was piloted in Phuoc Son from 29–31 March 2021 and was used throughout the full screening campaign in Tan Hiep. Eligible persons at these events, and particularly persons that did not present at the screening site and were subsequently visited at home, were recruited through convenience sampling.

### 2.6. Data Collection Procedures

**Figure 1:**
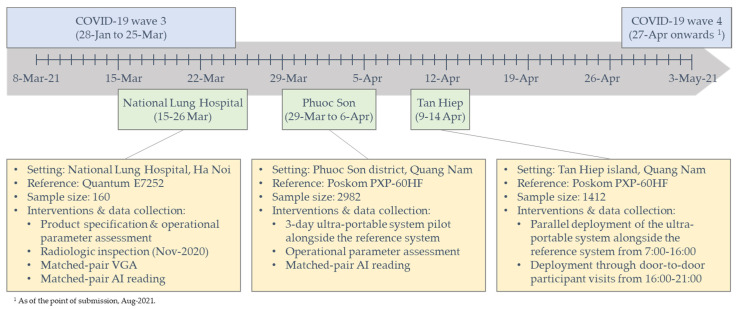
Illustration of study timeline, interventions and activities.

Figure 1 provides an illustration of the study timeline, field interventions and data collection activities. We recorded key product specifications and operating parameters of the system in comparison to those described in the user manual including weight, source-to-image distance (SID), exposure time, cycle time and battery life in terms of numbers of images taken with one charge. To assess safety, we commissioned a radiologic inspection of the X-ray generator by the Institute for Nuclear Science and Technology (INST) within the Viet Nam Atomic Energy Institute under the Ministry of Science and Technology. The assessment included 14 core parameters (Supplementary Materials, Table S1). To assess image quality, we conducted a basic visual grading analysis (VGA) [29] of the Xair system and the NLH’s reference X-ray system using a standard PA CXR from consenting participants. In addition to the standard PA CXR on the NLH’s reference X-ray system taken for their health-seeking purpose, these participants had a second CXR taken immediately after the reference CXR using the ultra-portable X-ray system by the same radiographer. The image quality was evaluated using a 5-point Likert scale (1: very low to 5: very high) by three radiologists of the NLH, including the head and deputy head of the radiology department, with qualifications including one master’s and two doctor of philosophy degrees and between 20–26 years of experience. Aside from the VGA by human readers, we processed the image pairs using qXR v3 (Qure.ai; Mumbai, India) AI software to yield quantitative scores on a scale of 0–1 for each CXR image for an unbiased measure of any differences in the ability to recognize abnormalities that are suggestive of TB.

Subsequently, we integrated the ultra-portable X-ray system into two community CXR screening campaigns. On these campaigns, key population groups vulnerable for active TB in the intervention district were invited to present for CXR screening at a designated date and screening site. If participants showed abnormalities on CXR suggestive of TB based on radiologist interpretation, their sputum was collected for testing with the Xpert MTB/RIF assay (Xpert). The head of the NLH’s radiology department remotely verified all abnormal CXRs within 24 hours of image capture. Additional implementation procedures and data collection methods for these mobile CXR screening events have been described elsewhere [30].

During each campaign, the ultra-portable system was used at the location of congregation during daytime hours (7:00 to 16:00). In the evenings, the ultra-portable system was used to conduct home visits for persons who were invited, but did not present at the designated screening sites, battery charge permitting. The reference X-ray system was used at the designated screening sites in parallel. When both systems were operating in parallel, individuals were assigned to the next available system. On the first three days in Phuoc Son, we conducted a comparative image quality analysis between the Xair system and the portable, outlet-powered device similar to the evaluation conducted at the NLH. These matched-pair images were similarly processed by the qXR software to obtain quantitative scores for each CXR.

### 2.7. Statistical Analyses

We conducted a side-by-side comparison of key operating parameters as described in the user manual and observed during use and reported the summary results from the external X-ray emissions inspection. For the VGA, we calculated the mean Likert scores for each radiologist and fitted fixed- and mixed-effect ordinal logistic regression models to explore the association between image quality rating and type of X-ray equipment. We calculated inter-rater reliability using Fleiss’ multi-rater Kappa statistic. Participant demographic and clinical covariates were secondary parameters, and the radiologist was the random effect in the multi-level model. We conducted an AI-supported post hoc analysis to determine potential confounding of the X-ray image interpretation on the VGA from suboptimal image properties on rotation, inspiration, positioning and exposure/penetration to detect bias due to a radiographer’s inconsistencies in obtaining the X-ray images [31]. The results of this adjusted analysis (Supplementary Materials, Table S2) showed virtually the same results as the initial VGA, so that we presented the initial analysis given the higher sample size. For the AI-based comparative analysis, we calculated the mean TB abnormality scores and fitted population-average logistic regression models using Generalized Estimating Equation (GEE) methods to measure the association between the abnormality score and X-ray equipment, while accounting for the inter-cluster differences of the two ACF sites. To enable convergence, models were specified with negative binomial distributions, log link functions and independent correlation structures based on a comparison of Quasi-Likelihood Information Criteria [32]. Secondary model parameters included participant age and sex, as well as covariates such as cough, fever, weight loss, night sweats, chest pain, dyspnea, fatigue, history of TB, diagnostic test results and screening site.

For the comparative yield analysis, we constructed TB care cascades disaggregated by X-ray equipment and calculated the yield and number needed to screen (NNS) [33]. We conducted a post hoc analysis of persons with TB detected among those screened at home by the ultra-portable X-ray device and calculated the NNS for this subgroup. Sample characteristics for participants of the community screening event are described in the Supplementary Materials (Table S3). We used Chi-squared tests to identify significant differences in each step of the cascade. Hypothesis tests were two-sided, point estimates included 95% confidence intervals and a p-value of less than 0.05 was considered significant. Statistical analyses were performed on Stata v17 (StataCorp; College Station, TX, USA).

### 2.8. Ethical Considerations

Ethical approvals were granted by the National Lung Hospital’s Institutional Review Board (10/20/CT-HDDD) and the Scientific and Ethical Committee of the Ha Noi University of Public Health (233/2021/YTCC-HD3). The study implementation was approved by the Ministry of Health (2742/QD-BYT) and the Quang Nam People’s Committee (390/QD-UBND). We obtained written informed consent from all participants and anonymized all patient data prior to analysis.

## 3. Results

Table 1 shows a comparison of system characteristics and key indicators between the values reported in the user manual of the ultra-portable X-ray system and its observed performance. On most of the metrics, both the generator and the flat-panel detector performed in line with values reported in the user manual. Particularly, the observed power-on time, time interval between exposures, transmission times from the detector to the workstation and battery charge time were comparable to the manufacturer’s reported values. We recorded a notable negative difference (−58%) in the observed capacity to capture images using a single battery charge (42 images vs. the reported 100 images). Conversely, the power-on and exposure cycle times of the detector were 33% and 43% faster than reported in the user manual, respectively.

We present the summary results of the ultra-portable X-ray system’s emissions verification report by the INST in Table 2. Similar to the above, the assessment concluded that the generator performed well within nationally permitted tolerances on all measured indicators such as peak voltage, exposure time, tube current, output dose, etc. Based on these emissions, the leakage dose at SIDs of 1 m and 2 m is reported to be between 0.0073–0.0136 µSv and 0.0028–0.0055 µSv at 90 KVp and 2.5 mAs, respectively.

Our VGA showed that the human radiologists perceived a significant difference in image quality (Table 3). In aggregate, the CXR taken by the stationary X-ray system received a mean rating of 3.99 (95% CI: [3.97, 4.01]), which was significantly higher (*p* < 0.001) compared to a mean rating of 3.71 (95% CI: [3.67, 3.76]) for the ultra-portable system. The results disaggregated by an individual radiologist showed a similar pattern of significantly lower ratings for the ultra-portable system compared to the reference. The multi-rater Kappa statistic for the VGA with three raters and two systems was 0.3144.

Table 4 shows the comparison of TB abnormality scores using AI software for both study settings, the NLH and the community ACF. Overall, we detected virtually no difference between the ultra-portable system and its two radiographic references. In the facility-based setting of the National Lung Hospital, the mean abnormality score from CXR images taken with the facility’s X-ray system was 0.48 (95% CI: [0.42, 0.54]). In comparison, the mean abnormality score from images taken with the ultra-portable X-ray system was 0.48 (95% CI: [0.42, 0.55]), indicating that there was no significant difference (*p* = 0.928). Similarly, the mean TB abnormality scores of images taken by the reference and the ultra-portable X-ray systems at the community-based TB ACF events were similar, at 0.20 (95% CI: [0.14, 0.27]) and 0.21 (95% CI: [0.14, 0.28]), respectively, also showing no significant difference (*p* = 0.377). Combining the samples from the two settings was concordant with individual results. Specifically, there was no significant difference (*p* = 0.571) in mean TB abnormality scores between the reference and the ultra-portable X-ray systems of 0.37 (95% CI: [0.32, 0.41]) and 0.37 (95% CI: [0.32, 0.42]), respectively.

**Figure 2:**
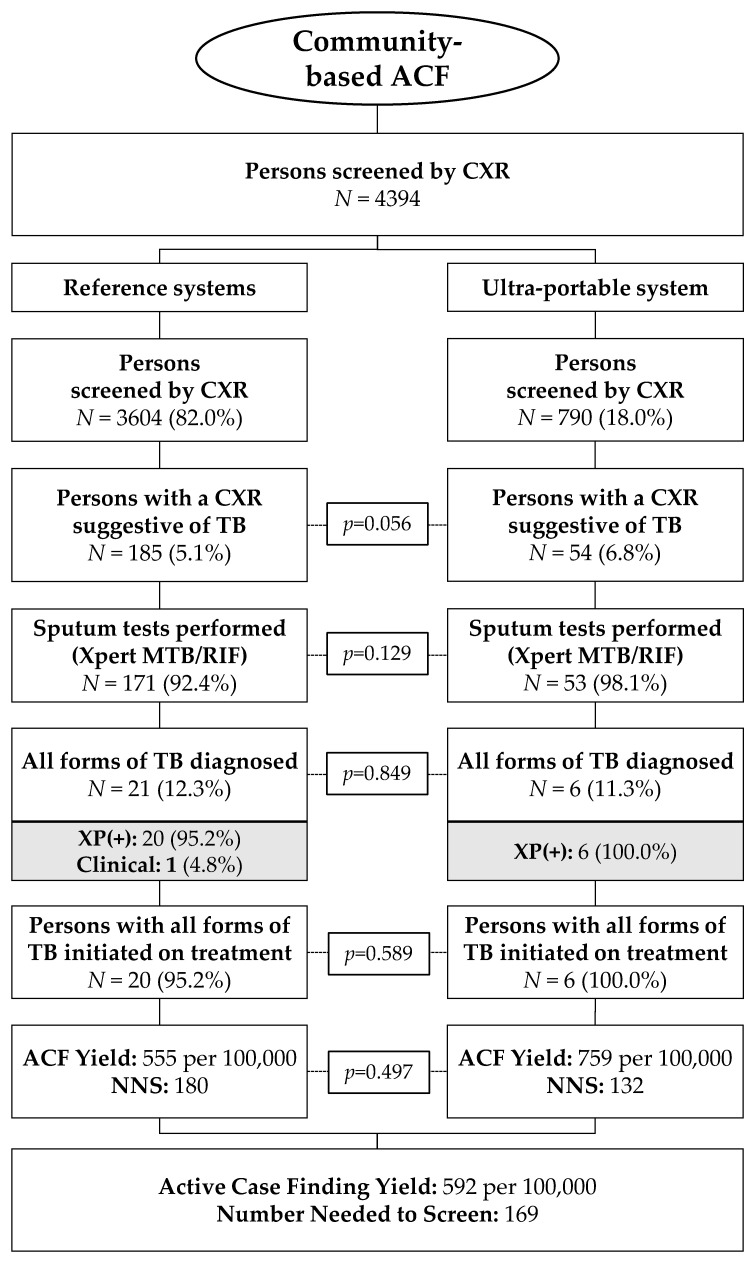
TB care cascade disaggregated by X-ray system used during the community screening events.

The TB care cascade from the two TB ACF campaigns disaggregated by reference and ultra-portable X-ray system is shown in Figure 2. In total, 4394 persons were screened by CXR, of whom 82.0% (3604/4394) were screened by the reference system and 18.0% (790/4394) by the ultra-portable system. The proportion of CXR images graded as having parenchymal abnormalities suggestive of TB by the on-site radiologist was not significantly different between the radiography systems (5.1% for the reference vs. 6.8% for the ultra-portable, *p* = 0.056). The rates of sputum collection and testing (85.1% vs. 84.1%), diagnosis of all forms of TB (12.3% vs. 11.3%) and linkage to care (95.2% vs. 100.0%) were similarly comparable between the reference and ultra-portable X-ray systems. The yield of TB patients linked to care from the combined ACF campaigns was 555 per 100,000 for the reference system for a NNS of 180 compared to a yield of 759 per 100,000 and a NNS of 132 for the ultra-portable X-ray system. The post hoc analysis in the subgroup of persons screened by the ultra-portable device at their homes (260/790 = 32.9%) showed a yield of two persons with TB linked to care for a yield of 769/100,000 and an NNS of 130.

## 4. Discussion

Our study showed that the ultra-portable X-ray system represents significant progress in the evolution of X-ray equipment that may be safely and effectively employed at the community and even household level, thereby extending X-ray access down to a greater proportion of vulnerable populations. In doing so, we surmise that the ultra-portable X-ray system employed in our study can be a highly useful tool in TB prevention and care, particularly in settings like Viet Nam that are relying on CXR as a critical component of the national diagnostic algorithm [34].

Specifically, we found that the system operated within the manufacturer’s reported emissions parameters and reported leakage doses that were well below the threshold doses for participants and health workers. With respect to the participants, the reported exposure and leakage doses were well below the average annual radiation dose from the environment (3 mSv) and the annual accepted dose of ionizing radiation for the general public (1 mSv) [11]. Regarding the health workers and especially the radiographers, the leakage doses were similarly below international guidelines on the stochastic limits for the occupational exposure of <20 mSv/year over five years [35]. Nevertheless, while the authorities authorized the community use for the ultra-portable X-ray system, the NLH also commissioned two radiography technicians to support the attending radiologist. To further assuage concerns, the system was positioned at opposite ends from the areas of congregation at the screening sites and the technicians wore a protective lead vest.

With respect to the ultra-portable radiography system’s utility for TB ACF and comparative field performance, we found no significant difference in its capacity to generate X-ray images that can elucidate recognizable abnormalities suggestive of TB. Both the AI-based comparative analysis and the proportions in the care cascade from the field deployment evinced that the proportion of TB abnormality and downstream case detection of the ultra-portable system were not inferior to the reference systems, despite a priori concerns over potentially impaired image quality. This suggests that there would be negligible impact on TB ACF yields despite operating outside of a health facility.

Instead, we recorded nominally higher CXR abnormality rates and case detection yields from the ultra-portable system. One potential explanation is that the system’s lightweight setup and significant improvement in mobility may have helped to reach a greater share of vulnerable persons during our community-based ACF campaigns in their homes. Numerous studies have shown that extending TB care closer to the homes of people affected by TB can result in greater case detection [36,37,38] and the provision of more people-centered care [39,40]. Thus, it is possible that there was a heterogeneity in TB prevalence among persons screened at the designated ACF screening sites and among persons in their homes screened by the ultra-portable system. However, given the lack of statistical evidence to substantiate this hypothesis, the lack discernable evidence in the sample characteristics and the high similarity in yield between the total cohort of persons screened by the ultra-portable system and those reached at their homes, more rigorous studies are needed to investigate this potential difference in utility and yield.

During our study, we identified a number of shortcomings that need to be overcome to optimize the use of these ultra-portable X-ray systems. Most critically, we found that the device was unable to capture the advertised number of images using one battery charge. This was particularly observed during the 3-day pilot in Phuoc Son, where we only managed to screen an average of 33 persons per day before the X-ray generator required recharging. To compensate for this shortcoming, we connected the system to a 40,000 mAh power bank for the Tan Hiep event, which ensured that we were able to power the device throughout the day. However, the manufacturers of these ultra-portable X-ray devices should strive to address this critical issue of throughput to improve utility and user experience.

While the power bank resolved the capacity issues, it in turn revealed another shortcoming in the X-ray generator’s inability to charge while turned on. This suggested that the device needed to be switched off between each image capture to charge the battery, so that screening could last throughout the day. Since using the power button on the device was cumbersome, a power strip with an on/off switch was used to accelerate the process. Even though this setup substantially improved the system’s usability and user experience, the ancillary equipment also impaired its portability. Additionally, the extra step in the operating procedure as well as the slightly elongated cycle time (adding ~5 s) between images were additional impairments of the user experience of the battery-operated system compared to the portable reference system that drew power directly from an outlet.

Another usability issue arose from the wireless transfer of images from the flat-panel detector to the processing station (i.e., laptop). While the study team readily accepted the slightly longer transmission time of 8 vs. 6 s, the main issue with the wireless image transmission pertained to the Bluetooth connectivity between the detector and the laptop. Specifically, these two devices disconnected on occasion during implementation in the field, which required the X-ray technician to carry the laptop closer to the detector to re-establish connectivity. This in turn interrupted the workflow and caused further undue delays, suggesting that future campaigns may utilize a wired connection for more reliable data transfer.

These process inefficiencies resulted in a scenario where the X-ray technicians relied exclusively on the outlet-powered reference system during periods of peak attendance at these ACF campaigns, in order to reduce participant wait times and prevent loss to follow-up.

Despite these shortcomings, the value proposition of these ultra-portable X-ray systems remains undeniable. As CXR triaging is firmly anchored in Viet Nam’s diagnostic roadmap [41], it also renders X-ray as the critical juncture that determines access to rapid molecular testing. Given the traditionally immobile nature of X-ray equipment, this reliance on CXR introduces new access barriers for health-seeking persons with TB, particularly in remote and hard-to-reach areas with low population densities such as Phuoc Son and Tan Hiep. In these settings, an ultra-portable CXR system may be an important catalyst to greater access to and equity in high-quality TB care.

As the objective of this study was to focus on the ultra-portable X-ray system’s utility for TB ACF rather than to optimize dose efficiency, a key limitation was that the procedures with which we evaluated image quality and safety lacked the sophistication of traditional radiographic evaluations. This was also evinced by the dichotomy in the lab evaluation between the VGA and TB abnormality threshold assessment. Specifically, the radiologists in our study recorded a significantly lower perceived image quality for the ultra-portable system compared to the reference system, possibly as a result of our use of a single composite image quality metric, which may have included factors unrelated to radiographic recognition of TB. A more rigorous VGA should have included a comprehensive set of image criteria [26,42], especially given the relatively low Kappa statistic. Concordantly, future evaluations should also entail contrast-detail curves and corresponding image quality figure inverse values from a technical phantom [43]. Thus, these evaluations may focus on optimizing the dose efficiency for these ultra-portable X-ray systems to align with the “as low as reasonably achievable” (ALARA) principle of radiography [44], particularly for persons vulnerable to TB, who are likely subjects for recurrent radiation exposure [45]. For the purposes of our study, we believe that the inclusion of an AI-based evaluation of image quality for TB abnormality sufficed to evince comparability in performance of the ultra-portable X-ray system for TB ACF and thereby mitigated the aforementioned methodological limitations.

A second limitation was the lack of precedence that could have informed our field deployment. We faced a steep learning curve with respect to the system’s field performance that precluded a more robust design and systematic integration of the ultra-portable system into TB ACF activities. Thus, the reproducibility of our methods and generalizability of our findings are limited. While we commissioned an external evaluation of the generator’s radiologic performance against reported specifications to assess adherence to radiation safety standards by proxy of emission rates, we were unable to measure leakage doses directly and subsequently relied principally on the manufacturer measurements for this metric. Lastly, the study was implemented in one of two months with no reported community transmission of SARS-CoV-2 in Viet Nam. As such, data presented in the TB care cascade are likely incomplete (and conservative), as social distancing and lockdowns impeded follow-up testing and completion of the clinical diagnostic algorithm.

Nevertheless, in recognition of the technological innovation and high potential to contribute to greater access to high-quality TB care of these ultra-portable X-ray systems, we believe our results can inform future studies and applications involving these devices, so their use can be optimized for faster, more equitable progress towards ending TB.

## 5. Conclusions

X-ray is a critical tool in the global effort to end TB. To overcome its pervasive access barriers, it is necessary to leverage technological advances such as the novel ultra-portable X-ray systems. Our study showed that, in spite of several areas in need of improvement, these systems have the potential to improve case detection and equitable access to high-quality TB care among the most vulnerable populations. As our study was exposed to a number of limitations, we strongly encourage further research and dissemination of implementation experiences to assess and maximize the utility and use of this new tool.

## Figures and Tables

**Table 1:** Key ultra-portable X-ray system characteristics and performance indicators as reported in the user manual and observed during use.

	Reported	Observed	Difference
**FDR XD2000 Xair**			
Weight [kg]	3.5	3.5	0%
Power-on time [s]	<120	<110	−8%
Time interval between exposures [s]	8	6	−25%
Max. image per battery charge [images] ^1^	100	42	−58%
Charge time from empty to full [hr]	4.5	4	−11%
Tube current [mA]	5	5	0%
Exposure time range [s]	0.04–0.5	0.04–0.5	−
Tube voltage range [kV]	50–90	50–90	−
Current time range [mAs]	0.2–2.5	0.2–2.5	−
Maximum load of shot number [shot/h] ^2^	200	180–200	−
**FDR D-EVO II G35**			
Power-on time [s]	<150	<100	−33%
Charge time from empty to full [h]	3	3	0%
Battery life [h]	3	3	0%
Preview image transmission time [s]	2	<2	0%
Image transmission time (wireless) [s]	7.5	8	7%
Image transmission time (wired) [s]	6	6	0
Exposure interval (wireless) [s]	9	9	0%
Exposure interval (wired) [s]	9	9	0%
Image storage capacity [DCM files]	100	100	0%
Exposure cycle time [s]	10.5	6	−43%
**Recommended operating conditions ^3^**			
Source-to-image distance [cm]	n/a	100	−
Tube voltage setting [kV]	n/a	90	−
Current time setting [mAs]	n/a	0.5	−

^1^ Exposure condition 90 kV, 0.5 mAs; time of lighting: 10 s; interval of the shots: 60 s; ^2^ exposure conditions of 90 kV, 0.5 mAs; 18 s. cycle; ^3^ for chest exposure.

**Table 2:** Summary results of the radiologic inspection of the ultra-portable X-ray generator.

	Measurement	Tolerance ^1^
**Peak kilovoltage accuracy [UkVp (%)]**		
50 kV	0.60%	±10%
70 kV	0.86%	±10%
90 kV	0.89%	±10%
**Peak kilovoltage reproducibility [R_kVp_ (%)]**		
80 kV	1.60%	±5%
**Exposure time [U_t_ (%)]**		
100 ms	0.50%	±20%
200 ms	0.30%	±20%
**Output dose reproducibility [R_L_ (%)]**		
80 kV and 10 mAs (100 mA * 100 ms)	1.71%	±20%
**Output dose linearity (%)**		
80 kV	4.39%	±20%
**Effective focal spot size**		
Effective focal spot size: 1.5 mm	25%	<40%
Nominal focus spot size: 1.2 mm		
**Perpendicularity of X-ray beam (°)**		
60 kV and 10 mAs	<1.50°	<1.50°
**X-ray to light field alignment (%)**		
Maximum deviation of one wall (60 kV and 10 mAs)	0.50%	<2%
Deviation of two walls in each axis (60 kV and 10 mAs)	0.50%	<3%
Deviation of all walls (60 kV and 10 mAs)	0.50%	<4%
**Primary beam filter (mmAl)**		
80 kV and 50 mAs	3.0	>2.3

^1^ As per national guidelines and provided by the Institute of Nuclear Science and Technology.

**Table 3:** Comparison of image quality by a human reader between the reference and ultra-portable X-ray systems at the National Lung Hospital.

	Reference X-ray System			Ultra-Portable X-ray System			
	N	Mean	95% CI	N	Mean	95% CI	*p*-Value ^1^
Radiologist #1	160	4.02	[3.98, 4.06]	160	3.63	[3.54, 3.71]	<0.001
Radiologist #2	160	3.97	[3.93, 4.01]	160	3.84	[3.78, 3.90]	0.001
Radiologist #3	160	3.99	[3.97, 4.02]	160	3.68	[3.60, 3.76]	<0.001
Overall	480	3.99	[3.97, 4.01]	480	3.71	[3.67, 3.76]	<0.001

^1^ Wald test from ordinal logistic regression for individual radiologists and mixed-effect ordinal logistic regression for the overall *p*-value adjusting for clinical and diagnostic covariates using robust standard error estimates.

**Table 4:** Comparison of mean AI scores of the ultra-portable and reference X-ray systems.

	Reference X-ray System			Ultra-Portable X-ray System			
	N	Mean	95% CI	N	Mean	95% CI	*p*-Value ^1^
National Lung Hospital	157	0.48	[0.42, 0.54]	157	0.48	[0.42, 0.55]	0.928
Community screening	108	0.20	[0.14, 0.27]	108	0.21	[0.14, 0.28]	0.377
Overall	265	0.37	[0.32, 0.41]	265	0.37	[0.32, 0.42]	0.571

^1^ Wald test from GEE population-averaged logistic regression model with a negative binomial distribution, log link function and an independent correlation structure with adjustment for clinical and diagnostic covariates as secondary model parameters using robust standard error estimates.

## Data Availability

Study data are property of the National TB Control Program and can be furnished upon reasonable request.

## Supplementary Materials

The following are available online at https://www.mdpi.com/article/10.3390/tropicalmed6030163/s1, Table S1: Testing parameters of the radiologic inspection of the X-ray generator by the Institute for Nuclear Science and Technology (INST), Table S2: Comparison of adjusted image quality by a human reader between the reference and ultra-portable X-ray systems at the National Lung Hospital, Table S3: Sample characteristics of participants in the community screening event.
