# Supplementary material for: Early Evaluation of an Ultra-Portable X-ray System for Tuberculosis Active Case Finding
Source: Trop Med Infect Dis. 2021 Sep 4;6(3):163. doi: 10.3390/tropicalmed6030163 (PMC8482270; doi:10.3390/tropicalmed6030163)
Supplement: Supplementary file 1 [file tropicalmed-06-00163-s001.zip › tropicalmed-1346467-supplementary.pdf]

SUPPLEMENTAL INFORMATION

Supplemental methods

Table S1. Testing parameters of the radiologic inspection of the x-ray generator by the Institute for Nuclear Science and Technology (INST).

Item	**Testing parameter**
1	High voltage waveform
2	Molding and appearance
3	Switches, buttons
4	Mechanical functionality, cassette tray, tube holder, tube crank, collimator
5	Distance indicator from tube focal spot to image receptor<br>Exposure alarm signal<br>Remote exposure controller
6	Peak kilovoltage accuracy
7	Peak kilovoltage reproducibility
8	Exposure time accuracy
9	Output dose reproducibility
10	Output dose linearity
11	Effective focal spot size
12	Perpendicularity of x-ray beam
13	x-ray to light field alignment
14	Primary beam filter (Half-value layer assessment)

Supplemental results

Table S2. Comparison of adjusted [1] image quality by a human reader between the reference and ultra-portable X-ray systems at the National Lung Hospital.

	Reference X-ray system			Ultra-portable X-ray system			
	**N [2]**	**Mean**	**95% CI**	**N**	**Mean**	**95% CI**	**p-value [3]**
Radiologist #1	134	4.02	[3.98, 4.06]	134	3.65	[3.55, 3.74]	<0.001
Radiologist #2	134	3.99	[3.95, 4.02]	134	3.82	[3.76, 3.89]	<0.001
Radiologist #3	134	4.01	[3.99, 4.04]	134	3.70	[3.62, 3.79]	<0.001 [4]
Overall	402	4.01	[3.99, 4.03]	402	3.73	[3.68, 3.77]	<0.001

[1] The adjusted VGA included images that were deemed comparable by AI on properties including rotation, inspiration, position and exposure/penetration using thresholds of 0.35, 0.8, 0.8-0.95 and 0.5-0.9, respectively, on a possible scoring range of 0 to 1; [2] 26 image pairs were found to have suboptimal image properties and were excluded from the adjusted VGA; [3] Wald test from ordinal logistic regression for individual radiologists and mixed-effect ordinal logistic regression for the overall p-value adjusting for clinical and diagnostic covariates with robust standard error estimates; [3] Wald test from ordinal logistic regression for individual radiologists with the covariate of night sweats omitted due to collinearity and unstable results of the saturated model.

Table S3. Sample characteristics of participants in the community screening event.

	**Reference X-ray system**	**Ultra-portable X-ray system**	**Total sample**	**p-value [1]**
Total participants [2]	3,604	790	4,394	
Age [median, IQR]	38 (27–52) (N=3,597)	43 (29–56) (N=789)	39 (27–53) (N=4,389)	<0.001
Sex [N, %]				
Male	1,655 (45.9%)	436 (55.2%)	2,091 (47.6%)	<0.001
Female	1,949 (54.1%)	354 (44.8%)	2,303 (52.4%)	
TB patient contact [N, %]				
No	3,368 (93.5%)	763 (96.6%)	4,131 (94.0%)	<0.001
Yes	236 (6.5%)	27 (3.4%)	263 (6.0%)	
SHI coverage [N, %]				
No	38/3,530 (1.1%)	7/781 (0.9%)	45/4,311 (1.0%)	0.654
Yes	3,492/3,530 (98.9%)	783/781 (99.1%)	4,266/4,311 (99.0%)	
Cough [N, %]				
No	2,915 (80.9%)	623 (78.9%)	3,538 (80.5%)	0.194
Yes	689 (19.1%)	167 (21.1%)	856 (19.6%)	
Fever [N, %]				
No	3,575 (99.2%)	789 (99.9%)	4,364 (99.3%)	0.036
Yes	39 (0.8%)	1 (0.1%)	30 (0.7%)	
Weight loss [N, %]				
No	3,547 (98.4%)	766 (97.0%)	4,313 (98.2%)	0.006
Yes	57 (1.6%)	24 (3.0%)	81 (1.8%)	
Night sweats [N, %]				
No	3,581 (99.4%)	784 (99.2%)	4,365 (99.3%)	0.703
Yes	23 (0.6%)	6 (0.8%)	29 (0.7%)	
Dyspnea [N, %]				
No	3,325 (92.3%)	778 (98.5%)	4,103 (93.4%)	<0.001
Yes	279 (7.7%)	12 (1.5%)	291 (6.6%)	
Chest pain [N, %]				

No	3,212 (89.1%)	765 (96.8%)	3,977 (90.5%)	<0.001
Yes	392 (10.9%)	25 (3.2%)	417 (9.5%)	
Appetite loss [N, %]				
No	3,516 (97.6%)	757 (95.8%)	4,273 (97.3%)	0.007
Yes	88 (2.4%)	33 (4.2%)	121 (2.7%)	
Fatigue [N, %]				
No	3,268 (90.7%)	733 (92.8%)	4,001 (91.1%)	0.060
Yes	336 (9.3%)	57 (7.2%)	393 (8.9%)	
History of TB [N, %]				
No	3,492/3,588 (97.3%)	774/786 (98.5%)	4,266/4,374 (97.5%)	0.060
Yes	96/3,588 (2.7%)	12/786 (1.5%)	108/4,374 (2.5%)	
BCG vaccination				
No	2,439/3,255 (74.9%)	585/785 (74.5%)	3,024 (74.9%)	0.813
Yes	816/3,255 (25.1%)	200/785 (25.5%)	1,016 (25.1%)	

[1] Chi-squared test for proportions and Wilcoxon rank-sum test for medians;

[2] Actual N size presented in the event of missing data.
